# Replicon typing of plasmids carrying *bla*_CTX-M-1_ in *Enterobacteriaceae* of animal, environmental and human origin

**DOI:** 10.3389/fmicb.2014.00555

**Published:** 2014-10-30

**Authors:** Katrin Zurfluh, Gianna Jakobi, Roger Stephan, Herbert Hächler, Magdalena Nüesch-Inderbinen

**Affiliations:** Institute for Food Safety and Hygiene, Vetsuisse Faculty, University of ZurichZurich, Switzerland

**Keywords:** plasmids, incompatibility groups, *bla*_CTX−M−1_, dissemination, food chain, human

## Abstract

**Objectives**: The aim of this work was to determine the plasmid replicon profiles of a collection of *bla*_CTX-M-1_-positive enterobacterial strains. The isolates originated from chicken in the production pyramid, healthy food-producing animals at slaughter (chicken, calves, and pigs), chicken retail meat, environmental isolates originating from water bodies, and isolates from humans. A selection of IncI and IncN plasmids were characterized by multilocus sequence typing in order to determine their epidemiological relatedness.

**Methods**: Transconjugants of 74 *bla*_CTX-M-1_-positive isolates were analyzed by PCR-based replicon typing and by PCR-based plasmid multilocus sequence typing.

**Results**: The incompatibility groups detected among the *bla*_CTX-M-1_-harboring plasmids included IncI1, IncN, IncHI1B, IncF, IncFIIS, IncFIB, and IncB/O, with plasmid lineage IncI1/ST3 predominating in isolates from chicken and from humans. Lineage IncN/ST1 was detected mainly in isolates from pigs. For the first time, *bla*_CTX-M-1_ genes encoded on IncHI1 plasmids were detected in isolates from cattle and from water bodies.

**Conclusions**: This study identifies plasmid lineages that are contributing to the dissemination of *bla*_CTX-M-1_ genes in the food chain, the environment, and humans.

## Introduction

Extended spectrum β-lactamases (ESBLs) are bacterial enzymes that catalyze the hydrolysis of the amide bond in ß-lactam ring of extended-spectrum cephalosporins (ceftazidime or cefotaxime) and monobactams (aztreonam) (Matagne et al., 1998). They represent the most important mechanism of antibacterial resistance in Gram-negative bacilli. Based on their primary sequence homology (Ambler et al., 1991) and their substrate profiles (Bush and Jacoby, 2010), ESBLs can be categorized into classes and groups, respectively, whereby the majority of ESBLs belong to Ambler class A and to the Bush group 2be.

Originally detected in human clinical isolates associated with nosocomial infections in the early 1990's (Paterson and Bonomo, 2005), the classical plasmid-mediated TEM- and SHV-ESBLs which derived from point mutations in the structural genes of their precursors TEM-1, TEM-2, and SHV-1, were predominant over the following decade. Their rapid spread within and between bacterial species promoted their dissemination beyond the hospital setting, with extensive use of cephalosporins in human medicine generally considered to be a major selective force. In the course of this development, over 300 TEM- and SHV-variants were and still continue to be detected. However, the last decade has seen a rapid displacement of these and other ESBLs by the universal dispersion of the CTX-M-family of enzymes which are cefotaximases that originated through mobilization of chromosomal *bla*_CTX−M_ genes from environmental *Kluyvera* spp. into mobile genetic elements, including transposons and plasmids (Cantón et al., 2012). The CTX-M enzymes are classified according to their amino acid similarities into the five major groups CTX-M-1, CTX-M-2, CTX-M-8, CTX-M-9, and CTX-M-25 (Naseer and Sundsfjord, 2011). To date, more than 150 different CTX-M sequence types have been identified and added to the Lahey database that provides a comprehensive overview of the currently known ESBLs (www.lahey.org/studies/). In recent years there has been an alarming spread of CTX-M-enzymes throughout the human population, food animals, wildlife and the environment (Cantón et al., 2012).

Although CTX-M groups vary in different parts of Europe, the most prevalent is CTX-M group 1 which includes the enzymes CTX-M-1 and CTX-M-15. While CTX-M-15 appears to be the predominant variant in ESBL-positive human fecal flora (Geser et al., 2012b; Nicolas-Chanoine et al., 2012; Wickramasinghe et al., 2012), CTX-M-1 constitutes the most frequently reported ESBL subtype in the EU in *E. coli* originating from food-producing animals and foods (EFSA, 2011).

Horizontal gene transfer via plasmids represents a key mechanism by which resistance genes disseminate among different bacterial populations. Therefore, monitoring the spread of plasmids is useful to follow the transmission of antimicrobial resistance genes from different environments, with PCR-based replicon typing procedures acknowledged as a useful method (Carattoli et al., 2005). The predominant plasmid replicon types found in antibiotic resistant *Enterobacteriaceae* isolated from humans and animals include incompatibility (Inc) groups F, A/C, L/M, I1, HI2, and N (Carattoli, 2009). Different *bla*_CTX−M−1_ types are associated with particular plasmid replicon types (Naseer and Sundsfjord, 2011). *bla*_CTX−M−1_ have frequently been reported on broad host-range replicon plasmids IncI and IncN, which appear to have a reservoir in animals with a high prevalence among *E. coli* of the avian fecal flora (Johnson et al., 2007).

The aim of this work was to determine the plasmid replicon profiles of a collection of *bla*_CTX−M−1_-positive enterobacterial isolates originating from broilers, cattle, and pigs at slaughter, chicken retail meat, environmental isolates originating from water bodies, and isolates from humans. Furthermore, a selection IncI and IncN plasmids were characterized by multilocus sequence typing in order to investigate their epidemiological relatedness.

The results presented in this study offer a contribution to the understanding of the pathways of *bla*_CTX−M−1_ genes through food-producing animals, aquatic environments and humans.

## Material and methods

### Collection of strains

#### Longitudinal sampling at broiler chicken farms

Three broiler farms distributed throughout Switzerland were investigated through the production pyramid between July 2013 and May 2014. Boot sock samples of the parental broiler breeder flocks were obtained according to the Swiss guidelines for sampling broiler chicken houses. Meconium from one-day-old euthanized broilers originating from the parent animals was obtained by expressing meconium from the cloacae into sterile bags. Boot sock samples were taken from broiler flocks originating from the same parent animals. Boot socks and the meconium were enriched for 24 h at 37°C in 250 and 10 ml of EE Broth (BD, Franklin Lakes, USA), respectively. Then, one loopful each of the enrichment cultures was streaked onto chromogenic Brilliance ESBL agar (Oxoid, Hampshire, UK) and incubated at 37°C for 24 h under aerobic conditions. Thereafter, colonies with different color and morphology were picked from the selective plates and sub-cultured on triple sugar iron-agar (TSI) agar (Oxoid) at 37°C for 24 h. Oxidase-negative isolates were subjected to identification by API ID 32 E (bioMérieux, Marcy l'Etoile, France). Susceptibility testing was performed using *E*-test strips, according to the manufacturer's instructions. The antibiotics tested were cefotaxime, ceftazidime, and cefepime alone and in combination with clavulanic acid. Isolates exhibiting an ESBL phenotype were screened by PCR for the presence of genes belonging to the *bla*_TEM_, *bla*_SHV_ and *bla*_CTX−M_ families, using primers described previously (Pitout et al., 1998; Geser et al., 2012a). Resulting amplicons were purified using the GenElute™ PCR Clean-Up (Sigma-Aldrich, Buchs, Switzerland) according to the manufacturer's recommendations. Custom-sequencing was performed by Microsynth (Balgach, Switzerland) and the nucleotide- and translated protein-sequences were analyzed with CLC Main Workbench 7.0.2 (CLC bio, Aarhus, Denmark). For database searches the BLASTN program of NCBI (http://www.ncbi.nlm.nih.gov/blast/) was used. One *bla*_CTX−M−1_-positive isolate per sample was selected for further characterization. A total of 20 *bla*_CTX−M−1_-positive isolates were collected for this study.

#### Samples from healthy chicken, cattle and pigs at slaughter

The strains originating from fecal samples of chicken, pigs and cattle were collected from healthy animals entering the slaughterhouses. They were screened for the occurrence of ESBL-producing *Enterobacteriaceae* in a study described previously (Geser et al., 2012a). From the resulting collection of strains, 32 *bla*_CTX−M−1_-positive isolates were selected for this study.

#### Samples from retail chicken meat

Strains obtained from poultry meat were investigated for the presence of *bla* genes in a recent study (Abgottspon et al., 2014). Thirteen isolates were available for this study.

#### Environmental samples

Eight *bla*_CTX−M−1_-positive strains isolated from rivers in Switzerland in 2012 (Zurfluh et al., 2013) were included in this study for further characterization.

#### Samples obtained from humans

The strains originating from fecal samples of healthy humans (10 isolates) or from fecal swabs of primary care patients (2 isolates) were detected in previous studies on fecal carriage rates of ESBL-producing *Enterobacteriaceae* in humans in Switzerland between 2010 and 2012 (Geser et al., 2012b; Nüesch-Inderbinen et al., 2013).

In total, 86 *bla*_CTX−M−1_-positive strains collected between 2009 and 2014 were available for further analysis. An overview of the isolates and their origins is given in Table S1.

#### Transfer of plasmids by conjugation

Conjugation experiments were performed with the plasmid-free recipient strain *E. coli* HK225 (streptomycin resistant, rifampin resistant) (Kayser et al., 1982). Single colonies of the donor and recipient strains were grown overnight separately in Luria Bertani (LB) broth (Difco Laboratories, Franklin Lakes NJ, USA) at 37°C. Subsequently, equal volumes of the donor and recipient cultures were mixed and incubated overnight at 37°C without shaking. Serial dilutions were then plated on LB agar (Difco Laboratories) supplemented with 600 μg/ml streptomycin (Sigma-Aldrich, Buchs, Switzerland), 100 μg/ml rifampicin (Sigma-Aldrich) and 10 μg/ml cefotaxime (Sigma-Aldrich).

#### Plasmid replicon typing

Plasmid incompatibility (Inc) groups were determined by PCR-based replicon typing (PBRT) using genomic DNA of the transconjugants as template and primers described previously (Carattoli et al., 2005; Villa et al., 2010). In brief, DNA was obtained by a standard lysis procedure. Amplification by PCR was performed with 18 pairs of primers recognizing FIA, FIB, FIC, HI1, HI2, I1-Iγ, L/M, N, P, W, T, A/C, K, B/O, X, Y, F, and FIIA in 5 multiplex- and 3 simplex-reactions. Resulting amplicons were visualized by gel electrophoresis on a agarose gel stained with ethidium bromide.

#### Plasmid multi-locus sequence typing

IncI1 and IncN plasmids were subtyped by PCR-based plasmid multilocus sequence typing (pMLST) using genomic DNA obtained from transconjugants as template and primers described previously (García-Fernández et al., 2008, 2011). Amplicons were purified using the GenElute™ PCR Clean-Up (Sigma-Aldrich, Buchs, Switzerland) and custom sequenced by Microsynth (Balgach, Switzerland). Sequence types (STs) were assigned using the PubMLST database (http://pubmlst.org/plasmid).

## Results

### Conjugation experiments

Each of the 86 *bla*_CTX−M−1_-positive enterobacterial isolates was tested for its ability to transfer the cefotaximase-phenotype by conjugation. Seventy-four isolates transferred the resistance marker to the susceptible *E. coli* HK225 recipient, with transfer rates (transconjugants per donor cell) ranging from 7.48 × 10^−6^ (for *Klebsiella pneumoniae* strain OW61E isolated from a river sample) to 4.41 × 10^−2^, in the case of strain calf 128. Table S1 shows a summary of the conjugation experiments and the transfer rates of selected isolates. All 74 transconjugants were subjected to further investigation as described below.

### Plasmid replicon typing

A summary of the replicons detected among the 74 transconjugants is given in Table 1. Overall, 7 different replicons were identified, whereby IncI1 was the predominant type. Other Inc groups included IncN, IncHI1B, IncF, IncFIIS, IncFIB, and IncB/O. Isolates originating from chicken revealed the highest number of IncI1 types. Of the 41 typed avian strains, 40 were positive for IncI1, and only one for IncB/O. Four strains tested positive for two replicon types. In all 4 cases these were combinations of I1 and FIB.

**Table 1:** **Summary of replicons typed in *bla*_CTX-M-1_-plasmids originating from chicken, cattle, pigs, humans and environmental samples**.

**Source and Host**	**Total no. of isolates**	**No. of samples typed**	**Incompatibility groups determined in transconjugants (n)**	**pMLST^d^ (n) [associated replicon type]**	**Reference for origin of isolates**
**CHICKEN**					
Boot sock samples of parental broiler breeders	14	13	I1 (13); FIB^a^ (1)	ST3 (3) [I1]	This study
Meconium from broilers	3	3	I1 (3); FIB^a^ (2)	ST3 (1) [I1]	This study
Boot sock samples of broilers	3	3	I1 (3)	ST3 (1) [I1]	This study
Fecal samples chicken at slaughter	15	12	I1 (11); B/O (1); FIB^a^ (1)	ST3 (10) [I1]	Geser et al., 2012a
Meat product	14	10	I1 (10); FIB^a^ (1)	ST3 (3) [I1]	Abgottspon et al., 2014
**CATTLE**					
Fecal sample calves at slaughter	9	7	I1 (1); HI1B (3); FIB (1); N (2)	ST1 (2) [N]; ST3 (1) [I1]	Geser et al., 2012a
**PIGS**					
Fecal sample pigs at slaughter	8	7	I1 (2); B/O^b^ (1); F^c^ (1); FIIS^b^ (1); N (5)	ST1 (3) [N]; ST3 (1) [I1]; ST7 (1) [I1]	Geser et al., 2012a
**ENVIRONMENTAL SAMPLES**					
Rivers in Switzerland	8	7	I1 (3); HI1B (1); N (3)	ST1 (3) [N]; ST3 (3) [I1]	Zurfluh et al., 2014
**HUMANS**					
Stool samples of healthy humans	10	10	I1 (7); B/O (2); N(1)	ST1 (1) [N]; ST3 (6) [I1]; ST145 (1) [I1]	Geser et al., 2012b
Fecal swabs of primary care patients	2	2	I1 (2)	ST3 (2) [I1]	Nüesch-Inderbinen et al., 2013

a *In all cases associated with IncI1 plasmids*.

b *Both associated with one IncN plasmid*.

c *Associated with an IncN plasmid*.

d *Plasmid multilocus sequence typing (pMLST)*.

When compared to the Inc types isolated from avian samples, replicon types from other animals and the aquatic environment proved to be more diverse. Four Inc-types were identified among the 7 typed isolates originating from cattle and these included IncHI1B (*n* = 3), IncN (*n* = 2), IncI1 (*n* = 1), and IncFIB (*n* = 1). Isolates recovered from 7 porcine samples were positive for IncN (*n* = 5) and IncI1 (*n* = 2). One strain typed positive for 2 replicons (IncN and IncF) and one for three replicon types (N, FIIS and B/O).

Three Inc types, IncN (*n* = 3), IncI1 (*n* = 3), and IncHI1B (*n* = 1), were detected from isolates cultured from river samples. Interestingly, in human isolates (*n* = 12), IncI1 predominated (*n* = 9). Two other replicon types, IncB (*n* = 2), and IncN (*n* = 1) were detected in isolates from healthy humans.

The replicon types of all the successfully typed transconjugants are listed in Table S1. A concise summary of the results is shown in Table 1.

### Plasmid multi-locus sequence typing

Thirty-one IncI1 and 9 IncN-positive plasmids from isolates of all animal, environmental and human origins were selected for plasmid multilocus sequence typing. Three pMLSTs were identified; ST1, ST3 and, ST145. All 9 IncN-positive plasmids were associated with ST1. All except one IncI1-positive plasmids were assigned to ST3. One plasmid belonging to IncI1 originating from a healthy human strain typed ST145. An overview of all the plasmid multilocus sequence typed strains is given in Table S1 and the results are summerized in Table 1.

## Discussion

The global emergence and unequaled spread of *bla*_CTX−M_ genes in *Enterobacteriaceae* constitutes a serious public health concern and raises questions regarding their highly successful dissemination and the pathways they take through food animals, the aquatic environment and humans.

IncI1/ST3 plasmids clearly play a key role in the dissemination of *bla*_CTX−M−1_. Our results are suggestive for transmission of *bla*_CTX−M−1_ through the food chain to humans and the environment and are also confirmative of previous reports classifying poultry and chicken meat as major reservoirs of *bla*_CTX−M−1_ (Leverstein-van Hall et al., 2011), and broiler farms as emission sources of ESBL-producing *E. coli* into the environment (Laube et al., 2013). Our data also show that poultry is not the only source of IncI1/ST3-driven dissemination of *bla*_CTX−M−1_, but that cattle and pigs contribute, albeit to a lesser extent, as well. The epidemic IncN/ST1 plasmid lineage is specific to the food chain (García-Fernández et al., 2011), and our findings in cattle and pigs confirm that these plasmids are circulating among food producing animals. Our results suggest however, that this lineage does not contribute to the prevalence *bla*_CTX−M−1_ in humans to the same extent as the IncI1/ST3 lineage.

We detected the IncHI subgroup IncHI1B3 in two *E. coli* and one *Citrobacter youngae* isolates originating from healthy calves, as well as in one *E. coli* from a water sample. This is of interest, since IncHI1 plasmids are found predominantly in *Salmonella enterica* subspecies *enterica* serovar Typhi and Paratyphi A (Phan and Wain, 2008). So far, IncHI1 plasmids harboring *bla*_CTX−M−1_ have been described only in *E. coli* isolated from horses (Dolejska et al., 2014). To our knowledge, this is the first report of *bla*_CTX−M−1_ carried by IncHI1B plasmids in cattle, as well as in an isolate of aquatic origin. Additional studies will be needed in order to further elucidate the importance of this plasmid group for the dissemination of resistance genes in the food chain. Furthermore, it has to be mentioned that not all the 86 *bla*_CTX−M−1_ harboring isolates transferred their plasmids in our mating experiments. It therefore cannot be excluded that further Inc groups might have gone undetected.

The use of cephalosporins in husbandry has often been implicated as selective pressure for the successful dissemination of ESBL genes in food producing animals, especially in the poultry production pyramid. However, in Switzerland, 82% of antibiotic therapeutics for food-producing animals consist of sulfonamides, penicillins, and tetracyclins, with total sales declining since 2008 by 21.1% and since 2011 by 8.3% (Büttner et al., 2013). These data correlate with data on the sales of veterinary antimicrobial agents from 25 EU countries (European Medicines Agency, 2013). Hence, the use of antibiotics alone does not suffice to explain the prevalence and dissemination of IncI1 plasmids or other *bla*_CTX−M−1_-encoding plasmids in the food chain. There is evidence that IncI1 plasmids are maintained in *E. coli* cells without antibiotic selection pressure with little or no fitness cost to the host (Fischer et al., 2014), and that additional plasmid-encoded elements are beneficial to the bacterial host, such as toxin/antitoxin systems that account for stable inheritance of the plasmids to daughter cells, or type IV pili which benefit invasion and adhesion of *E. coli* to the host gut (Carattoli, 2009). In the case of poultry, vertical transmission of IncI1 plasmids in the poultry production pyramid from nucleus poultry flocks (Zurfluh et al., 2014), and coprophagy play an important role in the recycling of resistance genes via feces and may account for the high persistence of ESBL in chicken, even in organic broiler flocks that are never treated with antimicrobials (Bortolaia et al., 2010). Interestingly, it has been demonstrated that CTX-M-1-producing *E. coli* applied on fields in manure survive in soil for at least one year (Hartmann et al., 2012). This illustrates the high capacity of these bacteria to survive under environmental conditions in the absence of selection pressure. Conclusively, other forces besides antibiotics need to be taken in consideration when studying the spread and maintenance of ESBL-producing *Enterobacteriaceae* in animals, humans, and the environment. Besides decreasing antimicrobial use in animal production, additional measures to control resistance dissemination such as increased biosecurity in farms, and controls of animal origin and trade are of importance. Knowledge of the plasmid types circulating in bacterial populations is essential to advance new perspectives to control these plasmids, such as the development of replicon-targeted compounds, an antiplasmid approach suggested by DeNap and colleagues (DeNap and Hergenrother, 2005). This study describes the major plasmid lineages that are currently contributing to the dissemination of *bla*_ctx−m−1_ genes in the food chain and the environment in Switzerland. The plasmid lineage Inc1/ST3, which is predominant in chicken and chicken meat, is shared by human isolates harboring *bla*_ctx−m−1_. Chicken meat represents a major source of IncI plasmids in the food chain. A strategy to prevent the dissemination of these particular plasmids needs to be devised in future.

### Conflict of interest statement

The authors declare that the research was conducted in the absence of any commercial or financial relationships that could be construed as a potential conflict of interest.
