# Supplementary material for: Replicon typing of plasmids carrying blaCTX-M-1 in Enterobacteriaceae of animal, environmental and human origin
Source: Front Microbiol. 2014 Oct 30;5:555. doi: 10.3389/fmicb.2014.00555 (PMC4214192; doi:10.3389/fmicb.2014.00555)
Supplement: Supplementary file 1 [file Table1.DOCX]

**Table S1** Overview of the isolates containing CTX-M-1-encoding plasmids analysed in this study.

| Host and Isolate^a^ | Source | conjugational transfer/transfer rate^b^ | Inc group(s) | pMLST^c^ | Reference for origin of isolates |
| --- | --- | --- | --- | --- | --- |
| *parental broiler breeders* |  |  |  |  | This study |
| HV67 | boot sock | + | I1 | ST3 |  |
| HV164 | boot sock | + | I1 | – |  |
| HV194.1 | boot sock | + | I1 | – |  |
| HV171.1 | boot sock | + | I1 | – |  |
| HV331.1 | boot sock | + | I1 | – |  |
| HV331.2 | boot sock | + | I1 | – |  |
| HV331.3 | boot sock | + | I1 | – |  |
| HV332.1 | boot sock | + | I1 | – |  |
| HV333.1 | boot sock | + | I1 | – |  |
| HV336.1 | boot sock | + | I1 | – |  |
| HV337.1 | boot sock | + | I1 | – |  |
| HV338.1 | boot sock | + | I1 | – |  |
| HV364.1 | boot sock | + / 4.22 x10^-3^ | I1 | ST3 |  |
| HV365.1 | boot sock | - | – | – |  |
|  |  |  |  |  |  |
| *One-day-old broilers* |  |  |  |  | This study |
| HV359.1 | meconium | + | I1, FIB | ST3 |  |
| HV366 | meconium | + | I1, FIB | – |  |
| HV369.1 | meconium | + | I1 | – |  |
|  |  |  |  |  |  |
| *broiler* |  |  |  |  | This study |
| HV399 | boot sock | + | I1 | – |  |
| HV403.1 | boot sock | + | I1 | ST3 |  |
| HV408.1 | boot sock | + | I1 | – |  |
|  |  |  |  |  |  |
| *chicken at slaughter* |  |  |  |  | Geser *et al*., 2012a |
| chicken17 | faecal sample | + / 2.47 x10^-4^ | I1 | ST3 |  |
| chicken31 | faecal sample | + | I1 | ST3 |  |
| chicken32 | faecal sample | + | I1 | ST3 |  |
| chicken35 | faecal sample | - | – | – |  |
| chicken47 | faecal sample | - | – | – |  |
| chicken49 | faecal sample | + | I1 | ST3 |  |
| chicken58 | faecal sample | + | I1 | ST3 |  |
| chicken59 | faecal sample | + | I1 | ST3 |  |
| chicken60 | faecal sample | + | I1 | ST3 |  |
| chicken74 | faecal sample | - | – | – |  |
| chicken84 | faecal sample | + | I1 | ST3 |  |
| chicken86 | faecal sample | + | I1 | ST3 |  |
| chicken88 | faecal sample | + | I1 | ST3 |  |
| chicken87 | faecal sample | + | I1, FIB | – |  |
| chicken92 | faecal sample | + / 4.9 x10^-4^ | B/O | – |  |
|  |  |  |  |  |  |
| *chicken meat* |  |  |  |  | Abgottspon *et al*., 2014 |
| PB1 | meat | + | I1 | ST3 |  |
| PB5 | meat | + | I1 | – |  |
| PB7 | meat | - | – | – |  |
| PB8 | meat | - | – | – |  |
| PB10 | meat | + | I1 | – |  |
| PB11 | meat | + | I1 | – |  |
| PB15 | meat | - | – | – |  |
| PB21 | meat | + / 8.56 x10^-5^ | I1 | ST3 |  |
| PB22 | meat | + | I1 | – |  |
| PB24 | meat | + | I1 | – |  |
| PB29 | meat | + | I1, FIB | – |  |
| PB31 | meat | + | I1 | ST3 |  |
| PB34 | meat | + | I1 | – |  |
| PB35 | meat | - | – | – |  |
|  |  |  |  |  |  |
| *Cattle at slaughter* |  |  |  |  | Geser *et al*., 2012a |
| calf46 | faecal sample | + | HI1B | – |  |
| calf68 | faecal sample | + | N | ST1 |  |
| calf104 | faecal sample | - | – | – |  |
| calf112 | faecal sample | + | HI1B | – |  |
| calf128 | faecal sample | + / 4.41 x10^-2^ | I1 | ST3 |  |
| calf129 | faecal sample | + | FIB | – |  |
| calf136 | faecal sample | + | N | ST1 |  |
| calf142.09g^a^ | faecal sample | + | HI1B | – |  |
| calf192.09b | faecal sample | - | – | – |  |
|  |  |  |  |  |  |
| *Pigs at slaughter* |  |  |  |  | Geser *et al*., 2012a |
| pig13 | faecal sample | + | N | ST1 |  |
| pig14 | faecal sample | - | – | – |  |
| pig17 | faecal sample | + | N, F | – |  |
| pig18 | faecal sample | + | N | ST1 |  |
| pig60 | faecal sample | + | N, FIIS, B/O | – |  |
| pig64 | faecal sample | + / 3.02x10^-4^ | I1 | ST3 |  |
| pig65 | faecal sample | + / 1.32 x10^-2^ | I1 | ST7 |  |
| pig72 | faecal sample | + / 3.13 x10^-4^ | N | ST1 |  |
|  |  |  |  |  |  |
| *Environmental samples* |  |  |  |  | Zurfluh *et al.*, 2014 |
| OW10E2 | river sample | + | I1 | ST3 |  |
| OW29E | river sample | - | – | – |  |
| OW37E1 | river sample | + | N | ST1 |  |
| OW48E1 | river sample | + | HI1B | – |  |
| OW61E^a^ | river sample | + / 7.48 x10^-6^ | N | ST1 |  |
| OW63E1 | river sample | + | I1 | ST3 |  |
| OW65E2 | river sample | + | I1 | ST3 |  |
| OW68E2^a^ | river sample | + | N | ST1 |  |
|  |  |  |  |  |  |
| *Healthy humans* |  |  |  |  | Geser *et al.*, 2012 |
|  |  |  |  |  |  |
| HH1038 | stool sample | + | N | ST1 |  |
| HH1519 | stool sample | + | I1 | ST145 |  |
| HH1559 | stool sample | + | I1 | ST3 |  |
| HH1582 | stool sample | + / 6.71 x10^-4^ | I1 | ST3 |  |
| HH2018 | stool sample | + | I1 | ST3 |  |
| HH2238 | stool sample | + | I1 | ST3 |  |
| HH2290 | stool sample | + | I1 | ST3 |  |
| HH2291 | stool sample | + | I1 | ST3 |  |
| HH2332 | stool sample | + | B/O | – |  |
| HH2333 | stool sample | + | B/O | – |  |
|  |  |  |  |  |  |
| *Primary care patients* |  |  |  |  | Nüesch-Inderbinen *et al*., 2013 |
| HC192 | faecal swab | + / 4.38 x10^-4^ | I1 | ST3 |  |
| HC270 | faecal swab | + | I1 | ST3 |  |

^a^ all isolates were *Escherichia coli*, with the exceptions of calf 142.09 (*Citrobacter youngae*) and

OW61E1 and OW68E2 (both *Klebsiella pneumoniae*).

^b^ Transfer rate was determined per donor.

^c^ plasmid multilocus sequence typing (pMLST).

–; not determined.
